# Impact of intensive health education on influenza vaccination and acute exacerbations in outpatients with chronic obstructive pulmonary disease: a real-world study

**DOI:** 10.7189/jogh.15.04047

**Published:** 2025-03-07

**Authors:** Cong Liu, Qing Song, Ling Lin, Tao Li, Ping Zhang, Yuqin Zeng, Yating Peng, Yan Chen, Shan Cai, Ping Chen

**Affiliations:** 1 Department of Pulmonary and Critical Care Medicine, The Second Xiangya Hospital, Central South University, Changsha, China; 2 Research Unit of Respiratory Disease, Central South University, Changsha, China; 3 Clinical Medical Research Centre for Pulmonary and Critical Care Medicine in Hunan Province, Changsha, China; 4 Diagnosis and Treatment Center of Respiratory Disease in Hunan Province, Changsha, Hunan, China

## Abstract

**Background:**

The influenza vaccination rate of chronic obstructive pulmonary disease (COPD) patients in China was very low. In this study, we aimed to evaluate the effect of clinician-led intensive health education on influenza vaccination in outpatients with COPD and the effect of influenza vaccination on the risk of acute exacerbations in the real world.

**Methods:**

Participants were from the Real World Research of Diagnosis and Treatment of COPD study, a real-world prospective cohort study. COPD patients were included from December 2016 to April 2023 and followed up for one year. In January 2022, clinicians began strengthening health education for outpatients with COPD. We identified patients visiting the clinic from January 2022 to April 2023 as the intensive health education group and those visiting from December 2016 to December 2021 as the control group. We analysed factors associated with influenza vaccination and the effect of influenza vaccine on acute exacerbations by multivariate analysis.

**Results:**

7834 patients were included. Compared with the control group, the intensive health education group had a higher rate of influenza vaccination (1.6% *vs.* 12.2%, *P* < 0.01). Smoking cessation, high school education or above, influenza vaccination in the past year and intensive health education were independently associated with influenza vaccination. Influenza vaccination reduced the incidence of future acute exacerbations (adjusted odds ratio (aOR) = 0.48; 95% confidence interval (CI) = 0.33–0.68, *P* < 0.01), frequent acute exacerbations (aOR = 0.47; 95% CI = 0.27–0.82, *P* = 0.01), and severe acute exacerbation (aOR = 0.38; 95% CI = 0.23–0.63, *P* < 0.01) in COPD patients.

**Conclusions:**

Influenza vaccination reduced the risk of future acute exacerbations in patients with COPD. Clinician-led intensive health education can improve the influenza vaccination of outpatients with COPD, and clinicians and policymakers should pay attention to and apply this method.

Chronic obstructive pulmonary disease (COPD) is a common and preventable chronic airway disease characterised by persistent airflow restriction [1]. In China, the prevalence of COPD among people aged ≥40 years increased from 8.2% to 13.7%, and nearly 100 million people now suffer from the disease [2,3]. With the acceleration of the population’s ageing process, COPD will burden China’s public health care system. Therefore, preventing and treating COPD is an urgent matter.

The acute exacerbation of COPD is marked by accelerated lung function decline [4] and an increase in the risk of future hospitalisation and death [5]. Influenza is a major respiratory virus causing acute exacerbation of COPD [6]. The influenza vaccine, one of the most effective and cost-effective tools for preventing influenza, reduces the risk of acute exacerbation of COPD by 70% and reduces the risk of all-cause mortality in COPD patients [7,8]. Different COPD guidelines recommend influenza vaccination for COPD patients, especially elderly patients, as an important component of non-drug management of COPD [9–12].

However, influenza vaccination rates among COPD patients in China are extremely low. National cross-sectional studies have shown that influenza vaccination rates of COPD patients aged ≥40 years were 3.6% (95% confidence interval (CI) = 2.0–5.2) [13], while only 2.72% (95% CI = 2.34–3.10) were reported among hospitalised COPD patients [14]. This was far lower than the influenza vaccination rate in countries such as France (53.3%), Spain (68.3%), and the USA (66.3%) [15–17] and lower than the global average influenza vaccine coverage rate among people with chronic diseases (41.7%) [18]. In China, improving the influenza vaccination rate of patients with COPD is a top priority. In a cross-sectional survey, our research group found that the greatest obstacle to outpatient COPD patients receiving influenza vaccine was not understanding the importance of influenza vaccination [19]. Al-Qerem et al. found low influenza vaccination rates in Jordan, with forgetfulness and a lack of knowledge about vaccine effectiveness being the main barriers [20]. In Italy, one-third of the sample (32.9%) were unaware of vaccines recommended for their chronic medical conditions [21]. Health education is the most commonly used intervention to improve population vaccination [22]. In the real world, the effectiveness of intensive health education on influenza vaccination in outpatient COPD patients is unclear. Thus, the purpose of this study was to analyse the effect of intensive health education on influenza vaccination in outpatient COPD patients in the real world and to provide evidence for exploring the intervention mode of improving influenza vaccination in outpatient COPD patients.

## METHODS

### Study participants

Participants were enrolled in the Real World Research of Diagnosis and Treatment of COPD study (registration number: ChiCTR-POC-17,010,431), a real-world, prospective cohort study that had professional medical nurses to collect patient information and be responsible for the quality control of the data. The study followed patients every six months to record information on acute exacerbations, medication, vaccination, *etc.* [23]. We included outpatient COPD patients enrolled between December 2016 and April 2023. The diagnostic criteria for COPD were based on the Global Initiative for Chronic Obstructive Lung Disease (GOLD) 2017 report [1], with forced expiratory volume in one second (FEV1)/forced vital capacity <0.7 after bronchodilation. We excluded patients who were unable to communicate due to cognitive dysfunction, and patients with severe liver and kidney function and cardiac insufficiency. Ethics Institutional Review Committee of the Second Xiangya Hospital of Central South University approved the study (approval number 2016076). We conducted the study in accordance with the Declaration of Helsinki. We obtained a written informed consent from all participants.

### Study design

Early cross-sectional studies by our research group found that the main reason COPD outpatients did not receive an influenza vaccine was the lack of understanding of the importance of influenza vaccination [18]. To increase the influenza vaccination rate in outpatients with COPD, clinicians (researchers Dr Liu and Dr Song) began to implement intensive health education for outpatients with COPD on 1 January 2022. Dr Liu was responsible for the training and informed the trained doctors of the following key points: 1) during face-to-face communication, patients should be advised to receive the influenza vaccine, 2) health brochures should be issued to patients, and 3) in the third month after the visit, a unified text message should be sent to patients recommending influenza vaccination.

COPD patients who visited the clinic from January 2022 to April 2023 received intensive health education and we identified them as the intensive health education intervention group (intervention group). COPD patients who visited the clinic from December 2016 to December 2021 received general health education and we identified them as the control group. General health education consisted of clinicians briefly informing patients that they need regular medication, smoking cessation, and influenza vaccination, while intensive health education was based on general health education. First, during face-to-face communication with patients, we provided patients with detailed knowledge related to the influenza vaccine, such as the role of the vaccine, vaccination location, vaccination frequency, and protection time. Second, patients were issued COPD health management brochures, including the introduction of COPD characteristics, COPD risk factors, COPD drug treatment, COPD non-drug treatment, *etc.* Non-drug treatment of COPD included the 5A (ask, advise, assess, assist, arrange) model of quitting smoking and recommendations for influenza vaccination. Third, at the third month time point after the patient’s visit, we sent the content ‘COPD is a chronic airway disease; daily management includes regular inhalation of drugs, smoking cessation, flu vaccination, *etc.*’ to patients by text message.

### Data collection

Data collection included baseline data and follow-up data. We collected baseline data at the patient visit, including age, sex, marital status, education, smoking status, occupational exposure, lung function, the COPD assessment test score (CAT score), the modified Medicine Research Council (mMRC) dyspnoea scale score, acute exacerbations in the past year, and influenza vaccination of the past year. Further, we collected information on the number of acute exacerbations and influenza vaccination at a time point one year after the visit.

### Definition

We defined old age as ≥65 years. Further, we defined smoking cessation as quitting smoking for >6 months and non-smoking as having a cumulative smoking volume of <100 cigarettes and not currently smoking. According to the GOLD 2017 report [1], the GOLD group was divided into the GOLD group A, GOLD group B, GOLD group C, and GOLD group D according to the number of acute exacerbations in the past year, CAT, and mMRC. According to FEV1/estimated value of FEV1(FEV1%pred) after the use of a bronchodilator, FEV1%pred ≥50% was GOLD grade 1–2, FEV1%pred <50% was GOLD grade 3–4. We defined moderate acute exacerbations as an increase in respiratory symptoms requiring oral corticosteroids and/or antibiotics and severe acute exacerbations as an increase in symptoms requiring emergency department visits or hospital admission. Patients obtaining the influenza vaccination in the coming year were considered the influenza vaccination group.

### Statistical analysis

We performed statistical analysis using SPSS, version 26.0 (IBM, Armonk, NY, USA). We described quantitative variables as mean and standard deviation (SD) and qualitative variables as absolute quantities and percentages. Moreover, we used the Kolmogorov-Smirnov test to analyse the distribution of variables and in bivariate analysis, we used the Student’s *t* test to analyse normally distributed variables. Non-normally distributed variables were analysed by the Mann-Whitney U test. We used χ^2^ test to analyse qualitative variables. We considered a *P*-value <0.05 statistically significant. Further, we used multivariate logistic regression to calculate various adjusted odds ratios (aOR), and the commonality analysis was carried out before.

## RESULTS

### Information on all study subjects

**Figure 1:**
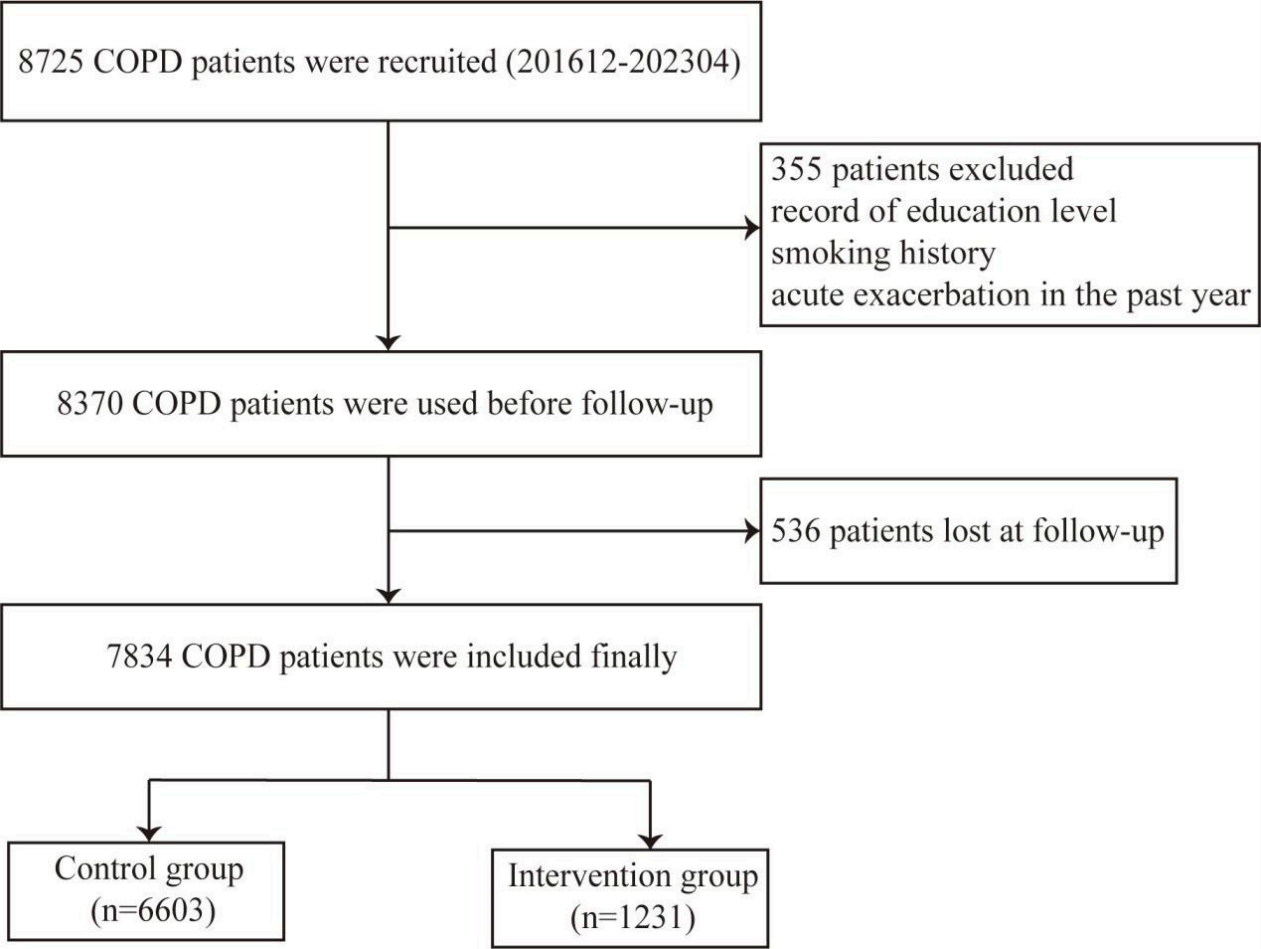
Flowchart for patient selection. COPD – chronic obstructive pulmonary disease.

In this study, a total of 7834 COPD patients were included (**Figure 1**), 85.9% were male, with an average age of 65.15 years (SD = 9.2), and FEV1%pred was 55.82 (SD = 21.1). The patients of GOLD group B and GOLD group D accounted for 36.8% and 38.8%, respectively. 56.6% of patients had acute exacerbations in the past year, and 75.7% of patients had CAT≥10. Compared with the control group, the proportion of intervention group patients aged ≥65 years, with primary school education level and below, GOLD grade 3–4, in the GOLD D group, with acute exacerbations in the past year, and with CAT≥10 was lower. The difference was statistically significant (**Table 1**).

**Table 1:** Clinical characteristics of patients with COPD*

Characteristics	Total	Control group	Intervention group	*P*-value
Total	7834	6603 (84.3)	1231 (15.7)	
Gender				
*Male*	6726 (85.9)	5863 (85.3)	1091 (88.6%)	
*Female*	1108 (14.1)	968 (14.7)	140 (11.4%)	
Age in years, x̄ (SD)	65.15 (9.2)	65.25 (9.3)	64.58 (8.4)	0.01
*<65*	3389 (43.3)	2800 (42.4)	589 (47.8)	<0.01
*≥65*	4445 (56.7)	3803 (57.6)	642 (52.2)	
Marital status				0.16
*Married*	7359 (93.9)	6213 (94.1)	1146 (93.1%)	
*Unmarried*	115 (1.5)	99 (1.5)	16 (1.3)	
*Widowed*	360 (4.6)	291 (4.4)	69 (5.6)	
Educational level				0.01
*Primary and below*	3236 (41.3)	2785 (42.2)	451 (36.6)	
*Junior high school*	2857 (36.5)	2389 (36.2)	468 (38.0)	
*Senior high school and above*	1741 (22.2)	1429 (21.6)	312 (25.3)	
Smoke status				0.07
*Never smoker*	1493 (19.0)	1281 (19.4)	212 (17.2)	
*Ex-smoker*	2786 (35.6)	2318 (35.1)	468 (38.0)	
*Current smoker*	3555 (45.4)	3004 (45.5)	551 (44.8)	
Occupational exposure				0.67
*No*	4544 (58.0)	3823 (57.9)	721 (58.6)	
*Yes*	3290 (42.0)	2780 (42.1)	510 (41.4)	
FEV1%pred, x̄ (SD)	55.82 (21.1)	55.35 (21.1)	59.08 (21.3)	<0.01
FEV1/FVC	48.63 (12.3)	48.47 (12.2)	49.73 (12.7)	<0.01
GOLD grades				<0.01
*1–2*	4497 (57.4)	3717 (56.3)	780 (63.4)	
*3–4*	3337 (42.6)	2886 (43.7)	451 (36.6)	
GOLD group				<0.01
*A†*	1312 (16.8)	1034 (15.7)	278 (22.6)	
*B*	2884 (36.8)	2456 (37.2)	428 (34.8)	
*C*	596 (7.6)	486 (7.4)	110 (8.9)	
*D†*	3042 (38.8)	2627 (39.8)	415 (33.7)	
Acute exacerbation in the past year				<0.01
*No*	3403 (43.4)	2812 (42.6)	591 (48.0)	
*Yes*	4431 (56.6)	3791 (57.4)	640 (52.0)	
CAT score				<0.01
*<10*	1906 (24.3)	1502 (23.0)	386 (31.4)	
*≥10*	5928 (75.7)	5083 (77.0)	845 (68.6)	
mMRC				<0.01
*0–1*	2753 (35.1)	2221 (33.6)	532 (43.2)	
*2–4*	5081 (64.9)	4382 (66.4)	699 (56.8)	
Drug therapy				<0.01
*LAMA†*	2072 (26.4)	1941 (29.4)	131(10.7)	
*ICS/LABA*	1117 (14.3)	944 (14.3)	173 (14.1)	
*LABA/LAMA†*	1015 (13.0)	680 (10.3)	335 (27.2)	
*ICS/LABA/LAMA*	3630 (46.3)	3038 (46.0)	592 (48.1)	

CAT – chronic obstructive pulmonary disease assessment test, COPD – chronic obstructive pulmonary disease, FEV1 – forced expiratory volume in one second, FEV1%pred – forced expiratory volume in one second/estimated value of FEV1, FVC – forced vital capacity, GOLD – Global Initiative for Chronic Obstructive Lung Disease, ICS – inhaled corticosteroid, LABA – long-acting β2-agonist, LAMA – long-acting, mMRC – modified medical research council dyspnoea scale, muscarinic antagonist, SD – standard deviation, x̄ – mean

*Presented as n (%) unless specified otherwise.

†There was a statistical difference between the two groups.

### The influenza vaccination and exacerbation status of COPD patients during one-year follow-up

**Figure 2:**
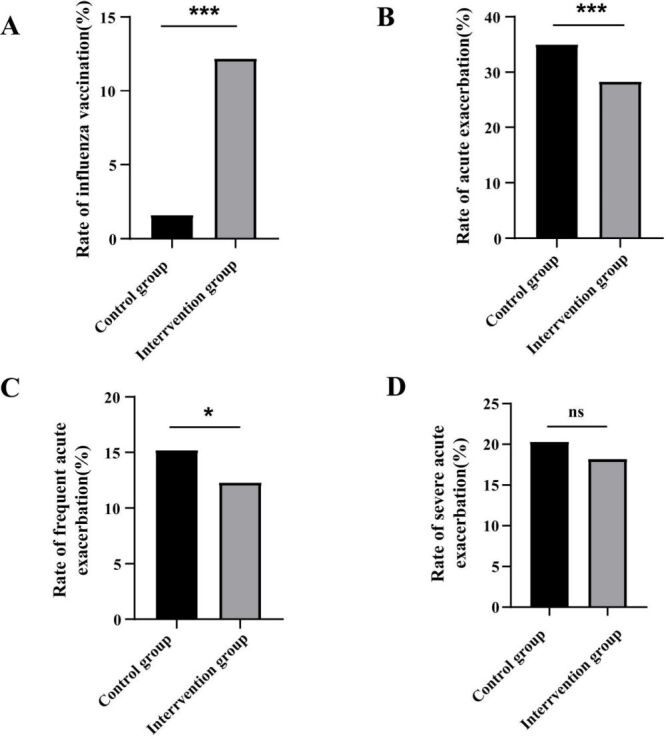
Influenza vaccine coverage rate and prognosis in patients with COPD between the control group and intervention group during one-year follow-up. **Panel A.** Rate of influenza vaccine between two groups. **Panel B.** Rate of acute exacerbation between two groups. **Panel C.** Rate of frequent acute exacerbation between two groups. **Panel D.** Rate of severe acute exacerbation between two group. *There was a statistical difference between the two groups.

**Figure 3:**
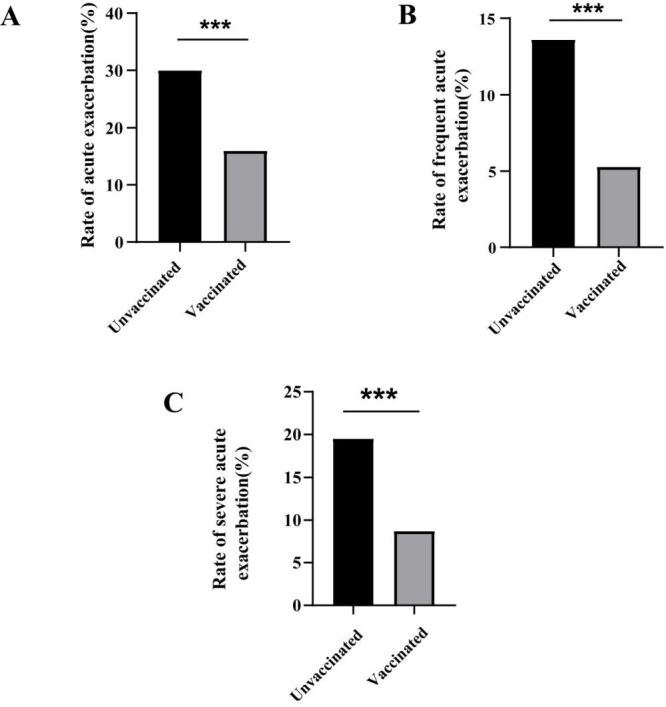
Prognosis of patients with influenza vaccine in intensive health education group during one year follow-up. **Panel A.** Rate of acute exacerbation between two groups. **Panel B.** Rate of frequent acute exacerbation between two groups. **Panel C.** Rate of severe acute exacerbation between two groups. *There was a statistical difference between the two groups.

As far as the control group is concerned, flu vaccination rates have not increased year over year since December 2016. Overall, influenza vaccination rates in the intervention group were significantly higher than those in the control group (1.6% *vs.* 12.2%, *P* < 0.01), and the rates of acute exacerbations (35.0% *vs.* 28.3%, *P* < 0.01) and frequent acute exacerbations (15.2% *vs.* 12.6%, *P* = 0.02) were lower. There was no difference between the rates of severe exacerbation in the two groups (**Figure 2**, **Table 2**). Through multivariate analysis, COPD patients receiving intensive health education intervention had a lower risk of future acute exacerbation (aOR = 0.79; 95% CI = 0.67–0.93, *P* = 0.01) and frequent exacerbation (aOR = 0.78; 95% CI = 0.62–0.98, *P* = 0.03) (**Table 3**). In the intensive health education group, the proportion of acute exacerbations (16.0% *vs.* 30.0%, *P* < 0.05), frequent exacerbations (5.3% *vs.* 13.6%, *P* < 0.05), and severe acute exacerbations (8.7% *vs.* 19.5%, *P* < 0.05), were significantly lower in vaccinated compared with unvaccinated patients (**Figure 3**).

**Table 2:** Influenza vaccine coverage rate and prognosis in patients with COPD after intensive health education during one-year follow-up*

Items	Control group	Intervention group	*P*-value
Rate of influenza vaccination			
*All*	107/6603 (1.6)	150/1231 (12.2)	<0.01
*December 2016–December 2017*	21/914 (2.3)	NA	
*January 2018–December 2018*	19/1504 (1.3)	NA	
*January 2019–December 2019*	25/1528 (1.6)	NA	
*January 2020–December 2020*	19/1345 (1.4)	NA	
*January 2021–December 2021*	23/1312 (1.8)	NA	
*January 2022–April 2023*	NA	150/1231 (12.2)	
Rate of acute exacerbation	2312/6603 (35.0)	348/1231 (28.3)	<0.01
Rate of frequent acute exacerbation	1002/6603 (15.2)	155/1231 (12.6)	0.02
Rate of severe acute exacerbation	1342/6603 (20.3)	224/1231 (18.2)	0.09

NA – not available

*Presented as n (%).

**Table 3:** Multivariate analysis of relative factors for exacerbation of COPD during one-year follow-up*

Variable	Acute exacerbation, aOR (95% CI)	*P*-value	Frequent acute exacerbation, aOR (95% CI)	*P*-value
Age in years				
*<65*	Ref.		Ref.	
*≥65*	1.06 (0.94–1.18)	0.36	0.90 (0.78–1.05)	0.18
Gender				
*Male*	Ref.		Ref.	
*Female*	0.92 (0.73–1.17)	0.49	0.84 (0.62–1.15)	0.28
Marital status				
*Married*	Ref.		Ref.	
*Unmarried*	0.73 (0.46–1.16)	0.19	0.67 (0.35–1.28)	0.23
*Widowed*	1.33 (1.04–1.71)	0.02	1.38 (1.01–1.89)	0.04
Educational level				
*Primary and below*	Ref.		Ref.	
*Junior high school*	0.89 (0.79–1.03)	0.08	0.88 (0.75–1.03)	0.12
*Senior high school and above*	0.96 (0.83–1.11)	0.57	0.97 (0.80–1.19)	0.77
Smoke status				
*Never smoker*	Ref.		Ref.	
*Ex-smoker*	1.09 (0.88–1.35)	0.43	1.02 (0.78–1.34)	0.88
*Current smoker*	0.98 (0.80–1.22)	0.91	0.90 (0.68–1.18)	0.45
Occupational exposure				
*No*	Ref.		Ref.	
*Yes*	1.17 (1.05–1.30)	0.01	1.48 (1.28–1.71)	<0.01
GOLD grade				
*1–2*	Ref.		Ref.	
*3–4*	1.11 (0.99–1.25)	0.08	1.20 (1.03–1.40)	0.02
GOLD group				
*A*	Ref.		Ref.	
*B*	1.05 (0.88–1.25)	0.58	1.28 (0.99–1.68)	0.06
*C*	2.26 (1.79–2.84)	<0.01	2.57 (1.87–3.55)	<0.01
*D*	2.32 (1.95–2.77)	<0.01	3.19 (2.47–4.12)	<0.01
Drug therapy				
*LAMA*	Ref.		Ref.	
*ICS/LABA*	1.03 (0.87–1.22)	0.76	1.09 (0.88–1.37)	0.43
*LABA/LAMA*	0.77 (0.64–0.94)	0.01	0.67 (0.52–0.88)	0.01
*ICS/LABA/LAMA*	0.86 (0.75–0.98)	0.03	0.82 (0.69–0.98)	0.03
Intervention group				
*No*	Ref.		Ref.	
*Yes*	0.79 (0.67–0.93)	0.01	0.78 (0.62–0.98)	0.03

aOR – adjusted odds ratio, CI – confidence interval, COPD – chronic obstructive pulmonary disease, GOLD – Global Initiative for Chronic Obstructive Lung Disease, ICS – inhaled corticosteroid, LABA – long-acting β2-agonist, LAMA – long-acting antimuscarinic agent, ref – reference

*Adjusted for age, gender, marital status, education level, smoke status, occupational exposure, GOLD grade, GOLD group, drug therapy, and intervention.

### Factors affecting influenza vaccination in COPD patients

Compared with the group that did not receive the influenza vaccine, the group that received the vaccine had a higher proportion of patients with high school education or above, smoking cessation, CAT<10, influenza vaccination in the past year, and patients receiving intensive health education (Table S1 in the **Online Supplementary Document**). Through multivariate analysis, smoking cessation (aOR = 1.50; 95% CI = 1.13–1.99, *P* = 0.01), high school education or above (aOR = 1.47; 95% CI = 1.06–2.05, *P* = 0.02), influenza vaccination in the past year (aOR = 6.18; 95% CI = 4.09–9.32, *P* < 0.01), and intensive health education (aOR = 8.86; 95% CI = 6.71–11.6, *P* < 0.01) were independently associated with influenza vaccination (Table S2 in the **Online Supplementary Document**). In the intensive health education group, patients with influenza vaccination in the past year and who have never been smokers or ex-smokers were more likely to get influenza vaccination (Figure S1 in the **Online Supplementary Document****)**.

### Effect of influenza vaccination on acute exacerbations in COPD patients over the next year

The rates of acute exacerbations (34.4% *vs.* 18.7%, *P* < 0.01), frequent acute exacerbations (15.0% *vs.* 7.0%, *P* < 0.01), and severe acute exacerbations (20.3% *vs.* 10.1%, *P* < 0.01) were lower in the vaccinated group than in the unvaccinated group (Table S3 in the **Online Supplementary Document**). After adjusting for age, sex, marital status, educational level, smoking status, occupational exposure, GOLD grade, GOLD grouping, and drug use, compared with unvaccinated COPD patients, COPD patients who received the influenza vaccine had lower risks of acute exacerbations (aOR = 0.48; 95% CI = 0.33–0.68, *P* < 0.01), frequent acute exacerbations (aOR = 0.47; 95% CI = 0.27–0.82, *P* = 0.01) and severe acute exacerbations (aOR = 0.38; 95% CI = 0.23–0.63, *P* < 0.01) (Table S4 in the **Online Supplementary Document**).

## DISCUSSION

Int this study, we found that intensive health education in the real world improved influenza vaccination and reduced the risk of acute exacerbation in outpatients with COPD. Clinical characteristics such as smoking cessation, educational level, and influenza vaccination in the past year were also independently related to influenza vaccination in COPD patients. Overall, influenza vaccination reduces the risk of future exacerbation among COPD patients. Intensive health intervention is a simple and effective approach, and clinicians and policymakers should pay attention to the role of this approach, which may be extended to other high-risk groups who need influenza vaccination.

Influenza vaccination was an important part of non-drug treatment for COPD patients. France (53.3%), Spain (62.7%), and the USA (66.3%) [15–17] had much higher flu vaccination rates than China. It was reported that 36.5% of hospitalised patients with COPD in Turkey had been vaccinated against influenza [24]. According to the Korean National Health and Nutrition Survey, up to 80% of elderly COPD patients in Korea have been vaccinated against influenza [25]. The higher rates of influenza vaccination in these countries may be related to systematic supportive vaccination policies, including guidelines for vaccination (vaccine candidate, priority target population, and timing of immunisation) and free vaccination policies, good health care infrastructure, and an adequate, affordable supply of prequalified vaccine [26]. In China, improving the influenza vaccination rate of COPD patients is an urgent problem and the starting point of this study.

Health education was the most used intervention to increase population vaccination, increasing vaccination rates by an average of 2.1 times [19]. The study found that clinician-led intensive health education increased the vaccination rates of outpatients with COPD by 8.86 (95% CI = 6.71–11.6) times in the real world. Intensive health education may also reduce hesitation about getting the flu vaccine and improve patients’ awareness, acceptance, and willingness to be vaccinated against influenza [27–31]. Overall, intensive health education reduced the risk of acute and frequent acute exacerbations in COPD patients. Compared with the control group, patients in the intervention group had less airflow restriction and a lower symptom burden, and the risk of future acute exacerbation may be lower. After further correcting these effects through multivariate analysis, this study still found that enhanced health education was an independent protective factor for future acute exacerbation, indicating that enhanced health education can reduce the risk of acute exacerbation in COPD patients because of increased overall flu vaccination rates. The incidence of severe acute exacerbations decreased in the intervention group but did not reach a statistical difference, which may be related to the low absolute number of influenza vaccinations in the intervention group. Further analysis in this study found that COPD patients in the intervention group could benefit from influenza vaccination, the proportion of severe acute exacerbations was significantly lower in vaccinated *vs.* unvaccinated patients (8.7% *vs.* 19.5%, *P* < 0.05).

This study also found that the risk of acute exacerbation, frequent acute exacerbation, and severe acute exacerbation were all lower in COPD patients receiving influenza vaccine than in those who did not. In multivariate analysis, influenza vaccination remained an independent protective factor for acute exacerbation in COPD patients. Yan et al. found that influenza vaccination could effectively reduce the risk of acute exacerbation and hospitalisation among COPD patients, with the highest efficacy in preventing acute exacerbation of COPD [7]. Gershon et al. [32] also found that seasonal influenza vaccination was significantly associated with a lower rate of laboratory-confirmed influenza-related hospitalisations. Kopsaftis et al. [33], by including multiple studies, found that influenza vaccination did not increase early disease deterioration in COPD patients but reduced the risk of long-term acute exacerbations, supporting the results of this study.

Although we found that intensive health education effectively improved influenza vaccination in outpatient COPD patients, it still did not achieve satisfactory results. Wang et al. [34] found that in the intervention of influenza vaccination for chronic disease patients in a Shanghai community, the influenza vaccination rate only increased by nearly 1% (1.73% *vs.* 0.75%), which also did not achieve the ideal effect. Intervention by text message reminders increased the influenza vaccination rate by 2.6%; in France, the vaccination rate increased by 1.01 times when doctors provided health education pamphlets; initiatives including television announcements, mailings, and the provision of lectures, case studies, *etc*., increased adult influenza vaccination rates (35% *vs.* 53%, *P* < 0.01) [35]. In addition, in China, the pay-it-forward intervention resulted in influenza vaccination rates ranging from 31.6% to more than 70% in the population [36]. The implementation of vaccines is complex and involves many aspects, including vaccine effectiveness, social development level, public health policy, insurance, convenience of vaccination, education level and awareness of vaccines [37]. The level of economic development was an important factor. A multicentre study found that influenza vaccine use was significantly higher in high-income countries than in low- and middle-income countries [38]. Second, public health policies were essential for improving influenza vaccination rates. Yang et al. [39] found that in China, a free national vaccination programme targeting key populations could increase its influenza vaccination coverage to 10, 20, 40, and 75% if its budget were increased to USD 344 million, USD 688 million, USD 1.377 billion, and USD 2.582 billion, respectively. In the future, covering influenza vaccine by national vaccination programmes may greatly increase the influenza vaccine uptake in COPD populations. Therefore, the promotion of the influenza vaccine in the COPD population should not be limited to intensive health education but should include multi-faceted efforts (free flu vaccination policy, including vaccination in routine COPD management, *etc.*).

In addition to intensive health education, we found that smoking cessation, having a high school education and above, and influenza vaccination in the past year were also independently associated with influenza vaccination in COPD patients, in agreement with previous investigators [13,40,41]. Clinicians need to be aware of the impact of these factors.

This study had some limitations. First, we used a historical cohort as the control group. As education can improve vaccination rates, we selected historical controls. To reduce the bias introduced by using historical controls, we selected the patients who visited the clinic from December 2016 to December 2021 as the control group, because their visit dates were closest to those of the intervention group. We have cited the low level of influenza vaccination in COPD patients over the past few years, which also supports the fact that health interventions can increase influenza vaccination rates. Compared with the intervention group, in the control group the proportion of patients with primary school education or below was higher, lung function was worse, and symptoms were more severe. However, using binary logistic regression corrected the influence of these factors. Second, factors such as income, employment, and insurance were associated with influenza vaccination. The limited data included in this study failed to explore the influence of these factors, which should be addressed in future research [13]. In addition, the vaccinations in this study were self-reported.

## CONCLUSIONS

Intensive health education, education level, smoking cessation, and influenza vaccination in the past year were strongly associated with future influenza vaccination in COPD patients. Intensive health education has improved influenza vaccination for outpatient COPD patients, effectively reducing the risk of future acute exacerbations, and clinicians and policymakers should pay attention to and apply this method.

## Additional material


Online Supplementary Document
