# Supplementary material for: Impact of intensive health education on influenza vaccination and acute exacerbations in outpatients with chronic obstructive pulmonary disease: a real-world study
Source: J Glob Health. 2025 Mar 7;15:04047. doi: 10.7189/jogh.15.04047 (PMC11886753; doi:10.7189/jogh.15.04047)
Supplement: Online Supplementary Document [file jogh-15-04047-s001.pdf]

Supplement to: Liu C, Song Q, Lin L, Li T, Zhang P, Zeng Y, Peng Y, Chen Y, Cai S, Chen P. Impact of intensive health education on influenza vaccination and acute exacerbations in outpatients with chronic obstructive pulmonary disease: a real-world study. J Glob Health. 2025;15:04047.

Figure S1. Influenza vaccine rate stratified by age, education level, and smoking status in intensive health education group*. **Panel A.** Rate of influenza vaccine between different age groups. **Panel B.** Rate of influenza vaccine between different smoking status groups. **Panel C.** Rate of influenza vaccine between different education level group. **Panel D.** Rate of influenza vaccine in patients with or without influenza vaccination in the past year. *There was a statistical difference between the two groups.

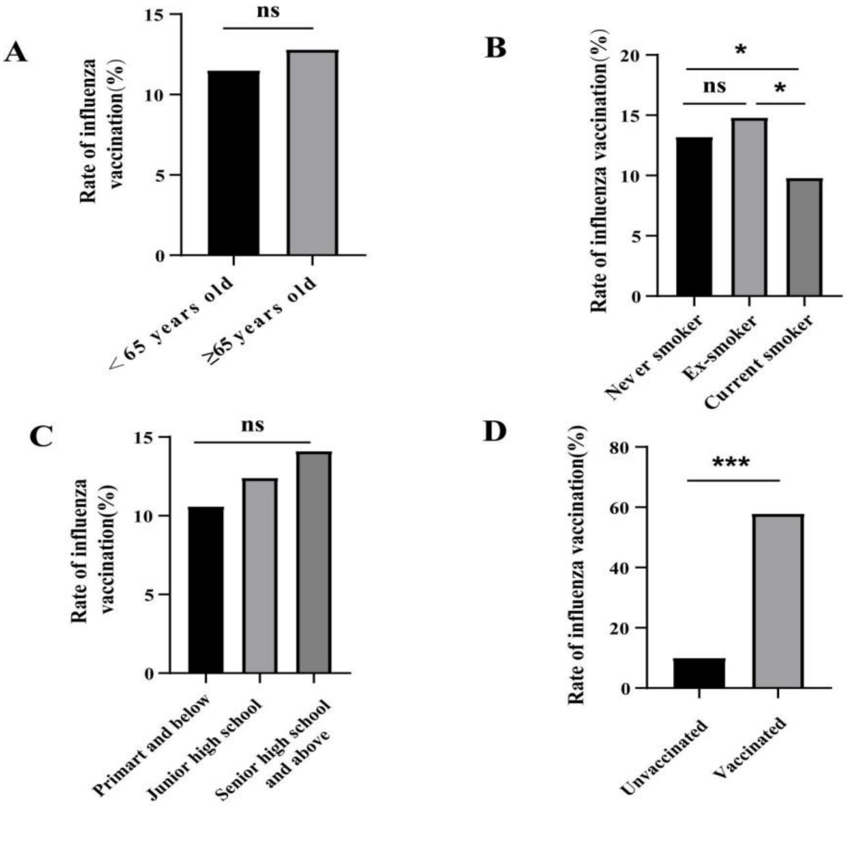

Table S1. Clinical characteristics of patients with COPD received influenza vaccine*

	Unvaccinated group	Vaccinated group	P value
Total	7577(96.7%)	257(3.3%)	
Gender			0.13
Male	6497(85.7%)	229(89.1%)	
Female	1080(14.3%)	28(10.9%)	
Age, years			
<65	3280(43.3%)	109(42.4%)	0.78
≥65	4297(56.7%)	148(57.6%)	
Marital status			0.07
Married*	7109(93.8%)	250(97.3%)	
Unmarried	114(1.5%)	1(0.4%)	
Widowed	354(4.7%)	6(2.3%)	
Educational level			0.04
Primary and below	3145(41.5%)	91(35.4%)	
Junior high school	2763(36.5%)	94(36.6%)	
Senior high school and above*	1669(22.0%)	72(28.0%)	
Smoking status			0.01
Never smoker	1452(19.2%)	41(15.9%)	
Ex-smoker	2671(35.2%)	115(44.7%)	
Current smoker	3454(45.6%)	101(39.4%)	
Occupational exposure			0.10
No	4382(57.8%)	162(63.0%)	
Yes	3195(42.2%)	95(37.0%)	
GOLD grades			0.20
1 to 2	4339(57.1%)	158(61.4%)	
3 to 4	3238(42.7%)	99(38.6%)	
GOLD group			0.05
A	1254(16.6%)	58(22.6%)	
B	2793(36.9%)	91(35.4%)	
C	573(7.6%)	22(8.6%)	
D	2955(39.0%)	86(33.5%)	
Acute exacerbation in the past year			0.05
No	3276(43.2%)	127(49.4%)	
Yes	4301(56.8%)	130(50.6%)	
CAT			0.01
<10	1826(24.1%)	80(31.1%)	
≥10	5751(75.9%)	177(68.9%)	
Intervention group			<0.01
No	6496(85.7%)	107(41.6%)	
Yes	1081(14.3%)	150(58.4%)	
Influenza vaccination in the past year			<0.01
No	7387(97.5%)	217(84.4%)	

Yes	190(2.5%)	40(15.6%)

Abbreviations: CAT – COPD assessment test; COPD – chronic obstructive pulmonary disease; FEV1 – forced expiratory volume in 1 second; FVC – forced vital capacity; GOLD – global initiative for chronic obstructive lung disease.

*There was a statistical difference between the two groups.

Table S2. Multivariate analysis of relative factors for influenza vaccination

Variable	aOR	95% CI	P value
Educational level			
Primary and below	Reference		
Junior high school	1.20	0.88-1.63	0.25
Senior high school and above	1.47	1.06-2.05	0.02
Smoke status			
Current smoker	Reference		
Never smoker	0.99	0.58-1.70	0.98
Ex-smoker	1.50	1.13-1.99	0.01
CAT			
<10	Reference		
≥10	0.83	0.62-1.11	0.20
Influenza vaccination in the past year			
No	Reference		
Yes	6.18	4.09-9.32	<0.01
Intervention group			
No	Reference		
Yes	8.86	6.71-11.6	<0.01

Note: Adjusted for education level, smoke status, CAT, intervention, influenza vaccination in the past year.

Abbreviations: CAT – COPD assessment test; aOR – adjusted Odds Ratio; 95% CI – 95% confidence interval.

Table S3. Prognosis of patients with COPD received influenza vaccine during one year follow-up

	Unvaccinated group	Vaccination group	P value
Rate of acute exacerbation, n (%)	2612/7577(34.4%)	48/257(18.7%)	<0.01
Rate of frequent acute exacerbation, n (%)	1139/7577(15.0%)	18/257(7.0%)	<0.01
Rate of severe acute exacerbation, n (%)	1540/7577(20.3%)	26/257(10.1%)	<0.01

Abbreviations: COPD – chronic obstructive pulmonary disease.

Table S4. Effect of influenza vaccination on acute exacerbation in patients with COPD by multivariate analysis

Variable	Acute exacerbation	P value	Severe acute exacerbation	P value	Frequent acute exacerbation	P value
Influenza vaccination						
No	Reference		Reference		Reference	
Yes	0.48(0.33-0.68)	<0.01	0.38(0.23-0.63)	<0.01	0.47(0.27-0.82)	0.01

Note: Adjusted for age, gender, marital status, education level, smoke status, occupational exposure, GOLD grade, GOLD group, drug therapy, influenza vaccination.

Abbreviations: COPD – chronic obstructive pulmonary disease; GOLD – global initiative for chronic obstructive lung disease; aOR – adjusted Odds Ratio; 95% CI – 95% confidence interval.
